# Raman spectroscopic study of cervical precancerous lesions and cervical cancer

**DOI:** 10.1007/s10103-020-03218-5

**Published:** 2021-01-06

**Authors:** Jing Wang, Cheng-Xia Zheng, Cai-Ling Ma, Xiang-Xiang Zheng, Xiao-Yi Lv, Guo-Dong Lv, Jun Tang, Guo-Hua Wu

**Affiliations:** 1 State Key Laboratory of PPTHIDCA/Department of Gynecology, The First Affiliated Hospital of Xinjiang Medical University, Urumqi, 830054 China; 2 College of Information Science and Engineering, Xinjiang University, Urumqi, 830046 China; 3 School of Electronic Engineering, Beijing University of Posts and Telecommunications, Beijing, 100876 China; 4 Physics and Chemistry Detecting Centre, Xinjiang University, Urumqi, 830046 China

**Keywords:** Cervical cancer, Cervical intraepithelial neoplasia, Cervix tissues, Raman spectroscopy, Support vector machine (SVM)

## Abstract

Early detection of cervical lesions, accurate diagnosis of cervical lesions, and timely and effective therapy can effectively avoid the occurrence of cervical cancer or improve the survival rate of patients. In this paper, the spectra of tissue sections of cervical inflammation (*n* = 60), CIN (cervical intraepithelial neoplasia) I (*n* = 30), CIN II (*n* = 30), CIN III (*n* = 30), cervical squamous cell carcinoma (*n* = 30), and cervical adenocarcinoma (*n* = 30) were collected by a confocal Raman micro-spectrometer (LabRAM HR Evolution, Horiba France SAS, Villeneuve d’Ascq, France). The Raman spectra of six kinds of cervical tissues were analyzed, the dominant Raman peaks of different kinds of tissues were summarized, and the differences in chemical composition between the six tissue samples were compared. An independent sample *t* test (*p* ≤ 0.05) was used to analyze the difference of average relative intensity of Raman spectra of six types of cervical tissues. The difference of relative intensity of Raman spectra of six kinds of tissues can reflect the difference of biochemical components in six kinds of tissues and the characteristic of biochemical components in different kinds of tissues. The classification models of cervical inflammation, CIN I, CIN II, CIN III, cervical squamous cell carcinoma, and cervical adenocarcinoma were established by using a support vector machine (SVM) algorithm. Six types of cervical tissues were classified and identified with an overall diagnostic accuracy of 85.7%. This study laid a foundation for the application of Raman spectroscopy in the clinical diagnosis of cervical precancerous lesions and cervical cancer.

## Introduction

Cervical cancer is the fourth most common cancer in the world in both morbidity and mortality, and the second most common cancer among women [1]. In some countries where screening systems for cervical cancer have been established, the incidence of cervical cancer has dropped by 65% in the past 40 years [2, 3]. However, in recent years, the incidence and mortality of cervical cancer in China have shown a significant upward trend, and the age of onset is trending younger [2, 3]. Cervical inflammation is common in women, and while chronic cervicitis is curable, long-term inflammatory cervical infections carry a 25% chance of inducing cervical cancer [4]. The occurrence of cervical cancer is a relatively slow process. Before developing into invasive cervical cancer, lesions must progress through the precancerous stage (CIN I, CIN II, CIN III). The precancerous stage may last for several years. Precancerous lesions of cervical cancer have a high potential to be cured. However, there are differences in treatment between cervical squamous cell carcinoma and cervical adenocarcinoma [2]. Therefore, early detection of cervical lesions, correct diagnosis of cervical lesions, and timely and correct treatment measures can effectively avoid the occurrence of cervical cancer or improve the survival rate of patients.

At present, the main screening methods for cervical precancerous lesions and cervical cancer are Thinprep cytologic test (TCT), human papillomavirus (HPV) screening, and combined TCT and HPV screening [1]. Colposcopy and biopsy are recommended for screening abnormal patients. In the past 30 years, the incidence of cervical squamous cell carcinoma has declined significantly in developed countries due to the cervical cancer screening project, but the incidence of cervical adenocarcinoma has increased. This may be due to the poor screening efficacy of cervical cytology screening methods for cervical adenocarcinoma [4]. In addition, these screening methods are time-consuming, costly, and invasive, so it is very important to find a rapid, economical, non-invasive, and objective diagnostic method for cervical lesions (including cervical adenocarcinoma).

Raman spectroscopy is a kind of inelastic scattering spectroscopy, which can quickly, objectively, and accurately detect slight differences between biochemical components of tissues. When used in combination with powerful multivariate algorithms, Raman spectroscopy can potentially provide automated, objective, and reproducible classification of pathology in clinically relevant time frames [5–7].

For example, Jess et al. used Raman spectroscopy to identify between primary human keratinocytes (PHK), PHK cells expressing the E7 gene of HPV-16 (PHK E7), and cervical cancer cells expressing HPV-16 (CaSki). This study was able to distinguish between normal keratinocytes and keratinocytes expressing HPV-16 E7, with a sensitivity and specificity of 93% and 93%, respectively [8]. Kim et al. used Raman spectroscopy to detect early HPV infection and cervical dysplasia without labels and achieved a better effect [9]. Duraipandian et al. explored the clinical application value of near-infrared Raman spectroscopy and genetic algorithms-partial least squares-discriminative analysis in colposcopy examination to identify the biomolecular changes in cervical tissues associated with malformation transformation. The established model in this study has a correct rate of 83% [10].

In this study, six types of cervical tissues were classified and identified by Raman spectroscopy combined with support vector machine (SVM) algorithm. Satisfactory diagnostic results were obtained. At present, an endoscopic Raman spectroscopy nasopharyngeal cancer detector has been developed to clinical application [11, 12]. It is believed that through further research, there is potential to develop a rapid, objective, economic, and non-invasive endoscopic Raman spectroscopy instrument for cervical lesion detection, which can be used clinically.

## Materials and methods

### Patients and ethics statement

The subjects of this study included cervical fluid–based cytology and/or high-risk HPV-positive patients who underwent cervical biopsy under colposcopy. A total of 210 patient samples were collected, including 60 cases of cervicitis, 30 cases of CIN I, 30 cases of CIN II, 30 cases of CIN III, 30 cases of cervical squamous cell carcinoma, and 30 cases of cervical adenocarcinoma. This study was approved by the ethics committee of Xinjiang Medical University, and the approval number is 20171123-12.

### Preparation of cervical tissue

Two hundred ten tissue sections from 210 patients (Among them, 60 were diagnosed as cervicitis, 30 were diagnosed as CIN I, 30 were diagnosed as CIN II, 30 were diagnosed as CIN III, 30 were diagnosed as cervical squamous cell carcinoma, and 30 were diagnosed as cervical adenocarcinoma by pathological diagnosis of the First Affiliated Hospital of Xinjiang Medical University.) were dewaxed and used in this study. The cervical tissue slices used in this study were prepared by professional doctors. The prepared tissue slices were stored in a dry and ventilated environment before dewaxing, and the preservation temperature was 25 ± 1 °C. The tissue slices after dewaxing were also stored in a dry and ventilated environment at a temperature of 25 ± 1 °C.

### Histopathological features

**Fig. 1:**
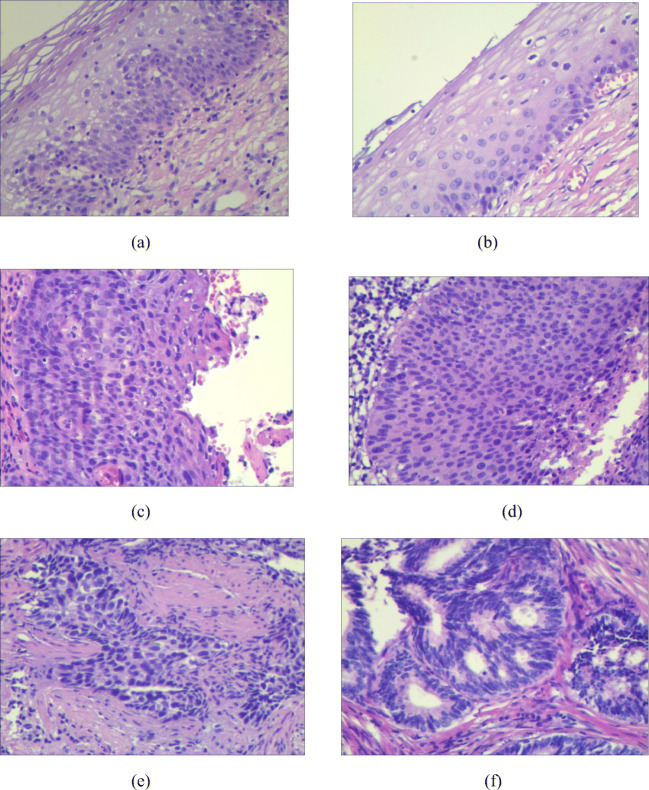
Representative micrographs of 6 types of tissues. **a** Photomicrograph of cervicitis tissue. **b** Photomicrograph of CIN I tissue. **c** Photomicrograph of CIN II tissue. **d** Photomicrograph of CIN III tissue. **e** Photomicrograph of cervical squamous cell carcinoma. **f** Photomicrograph of cervical adenocarcinoma

Figure 1 shows the representative micrographs of 6 types of tissues (cervicitis tissue, CIN I, CIN II, CIN III, cervical squamous cell carcinoma, and cervical adenocarcinoma). As can be seen from Fig. 1, the morphological differences of the six kinds of tissues are obvious and easy to distinguish. Specifically, Fig. 1a shows the cervicitis tissue, from which it can be seen that there were a large number of lymphocytes, plasma cells, and other chronic inflammatory cells in the cervical interstitium, which could be accompanied by hyperplasia of the cervical gland epithelium and interstitium and squamous metaplasia. Figure 1b shows the CIN I tissue, from which it can be seen that squamous basal and subbasal cell hyperplasia, mild dysplasia of nuclear polarity, mild atypia, few mitotic images, these cells confined to subepithelial 1/3 layer, P16 staining negative or spot-positive scattered in the epithelium. Figure 1c shows the CIN II tissue, from which it can be seen that the nuclear polarity was moderately disordered, with moderate atypia and increased mitotic images, and the heterotypic cells expand to the subepithelial 2/3 layer. Figure 1d shows the CIN III tissue, from which it can be seen that the nuclear polarity was completely disordered, the proportion of nuclear cytoplasm increased significantly, the mitotic images increased, and the heterotypic cells expanded to the whole subepithelial layer. P16 showed diffuse and continuous positive in more than two-thirds of the epithelial layer. Figure 1e shows the cervical squamous cell carcinoma tissue, from which it can be seen that infiltrating squamous cell carcinoma referred to the degree of cervical interstitial invasion beyond the microinvasive carcinoma, which mainly presented as a network or mass infiltration. There were three levels of differentiation: high (grade I), middle (grade II), and low (grade III) differentiation. Figure 1f shows the cervical adenocarcinoma tissue, from which it can be seen that there were three types, mucinous adenocarcinoma was the most common type, the tumor originated from the cervical mucous membrane columnar mucous cells, the gland structure could be seen under the microscope, and there were papillary processes in the gland cavity, glandular epithelial hyperplasia for multiple layers, low cells, obvious atypia, and increased mitosis in the nucleus.

### Raman spectral data acquisition

A confocal Raman micro-spectrometer (LabRAM HR Evolution, Horiba France SAS, Villeneuve d’Ascq, France) was used to record the spectrum. A 532-nm laser source was adopted, and the laser power was 25 mW. Raman spectra in the range of 400–1800 cm^−1^ were collected using a 50 (NA = 0.5) focal length lens. The integration time was 8 s, and the integration was performed three times.

### Statistical analysis

Four to eight spectra were collected from each tissue slice. Two hundred ninety-three spectra of cervical inflammation tissue, 157 spectra of CIN I tissue, 138 spectra of CIN II tissue, 155 spectra of CIN III tissue, 166 spectra of cervical squamous cell carcinoma tissue, and 201 spectra of cervical adenocarcinoma tissue were obtained, totaling 1110 spectra. An independent sample *t* test was used to analyze the difference of average relative intensity of Raman spectra of six types of cervical tissues. The level of significance was set at *p* ≤ 0.05. Then, the SVM algorithm was used to build an efficient diagnosis model to classify the six tissues. All procedure was implemented with MATLAB language.

## Results

### Raman spectroscopy

### Raman spectrum analysis

**Fig. 2:**
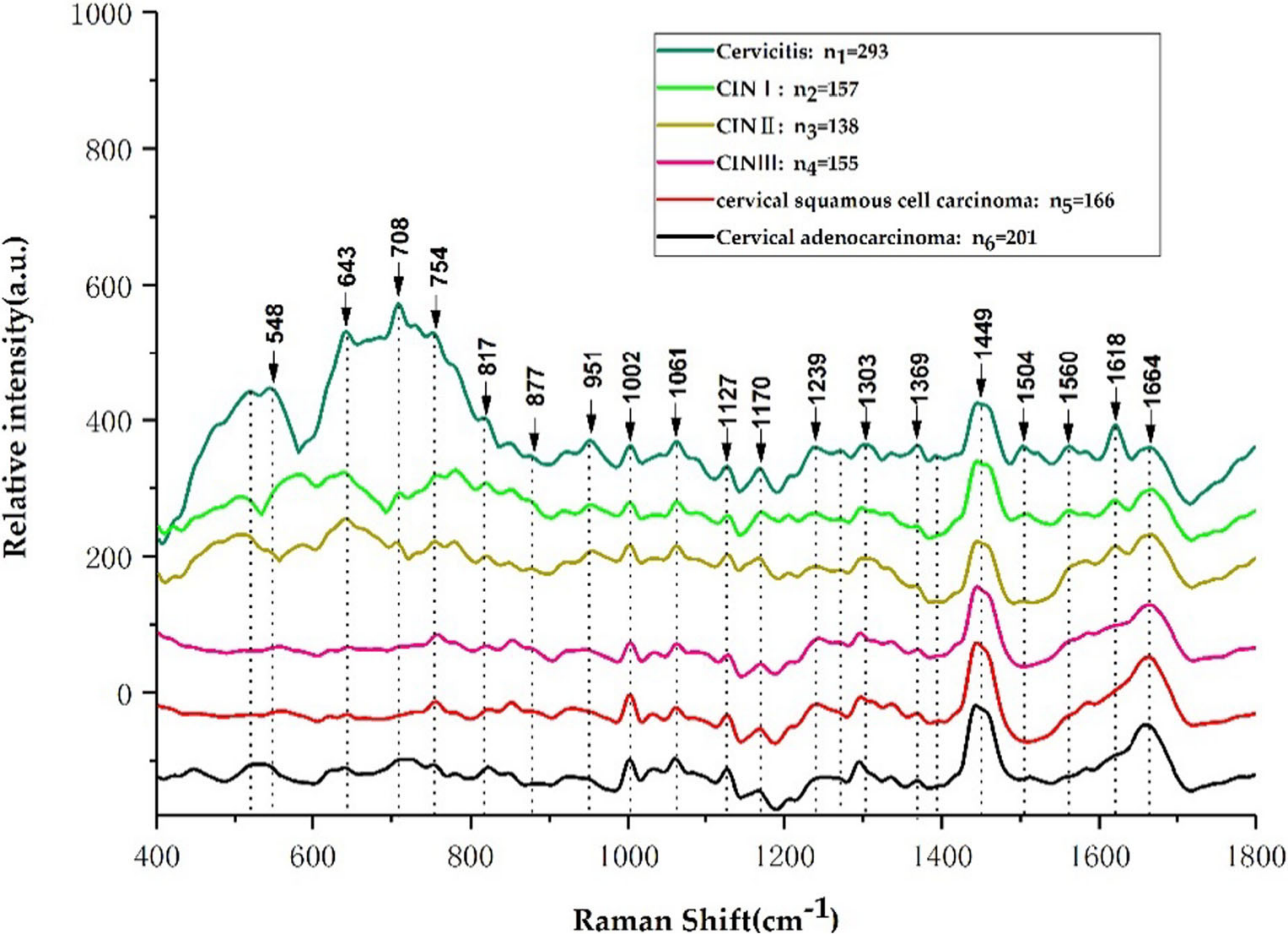
Average Raman spectra of 6 types of cervical tissues

As seen from Fig. 2, the Raman peaks of the average Raman spectra of the 6 types of cervical tissues mainly appear at 548, 643, 708, 745, 817, 877, 951, 1002, 1061, 1127, 1170, 1239, 1303, 1369, 1449, 1504, 1560, 1618, and 1664 cm^−1^. The Raman peaks at 745, 817, 877, 1002, 1061, 1127, 1170, 1239, 1369, 1449, and 1664 cm^−1^ are similar in appearance and in the same position in each sample. The Raman peaks in which there are differences in either shape or appearance or a combination of the two are primarily present at 548, 643, 708, 951, 1303, 1504, 1560, and 1618 cm^−1^, with obvious differences in Raman peaks at 519, 1270, and 1393 cm^−1^, marking clear differences in the Raman spectra of various cervical tissues. These differences are closely related to the corresponding changes in various biochemical components in cervical tissue with the development of cervical lesions.

**Fig. 3:**
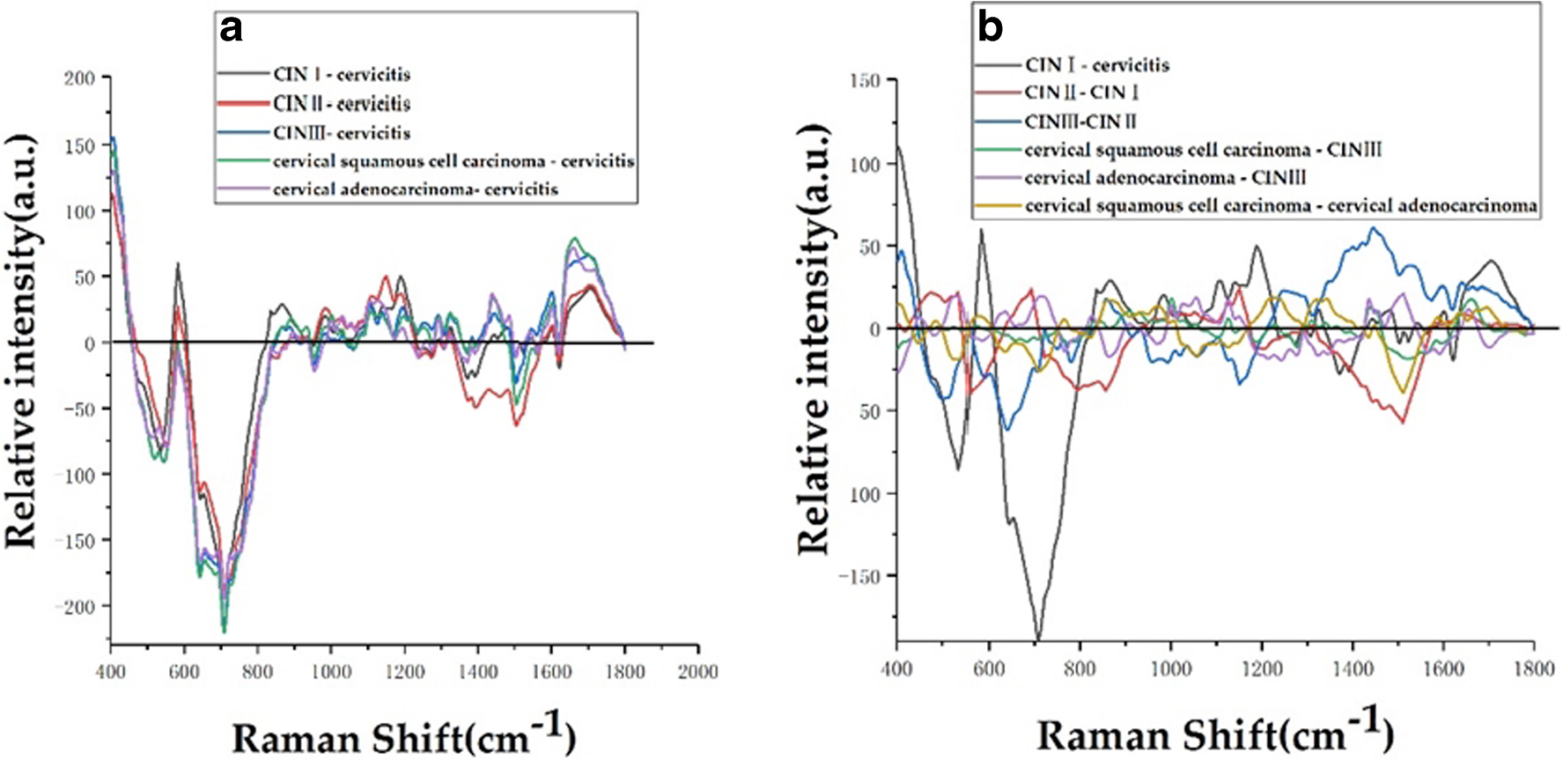
Average Raman spectral difference spectra of 6 types of tissues. **a** The difference spectrum of the average spectrum of CIN I, CIN II, CIN III, cervical squamous cell carcinoma, and cervical adenocarcinoma minus the average spectrum of cervical inflammation. **b** A plot of the following: CIN I mean spectrum - average spectrum of cervical inflammation, CIN II mean spectrum - CIN I mean spectrum, CIN III mean spectrum - CIN II mean spectrum, average spectrum of cervical squamous cell carcinoma - CIN III mean spectrum, average spectrum of cervical adenocarcinoma - CIN III mean spectrum, and the average spectrum of cervical squamous cell carcinoma - the difference spectrum of the average spectrum of cervical adenocarcinoma

**Fig. 4:**
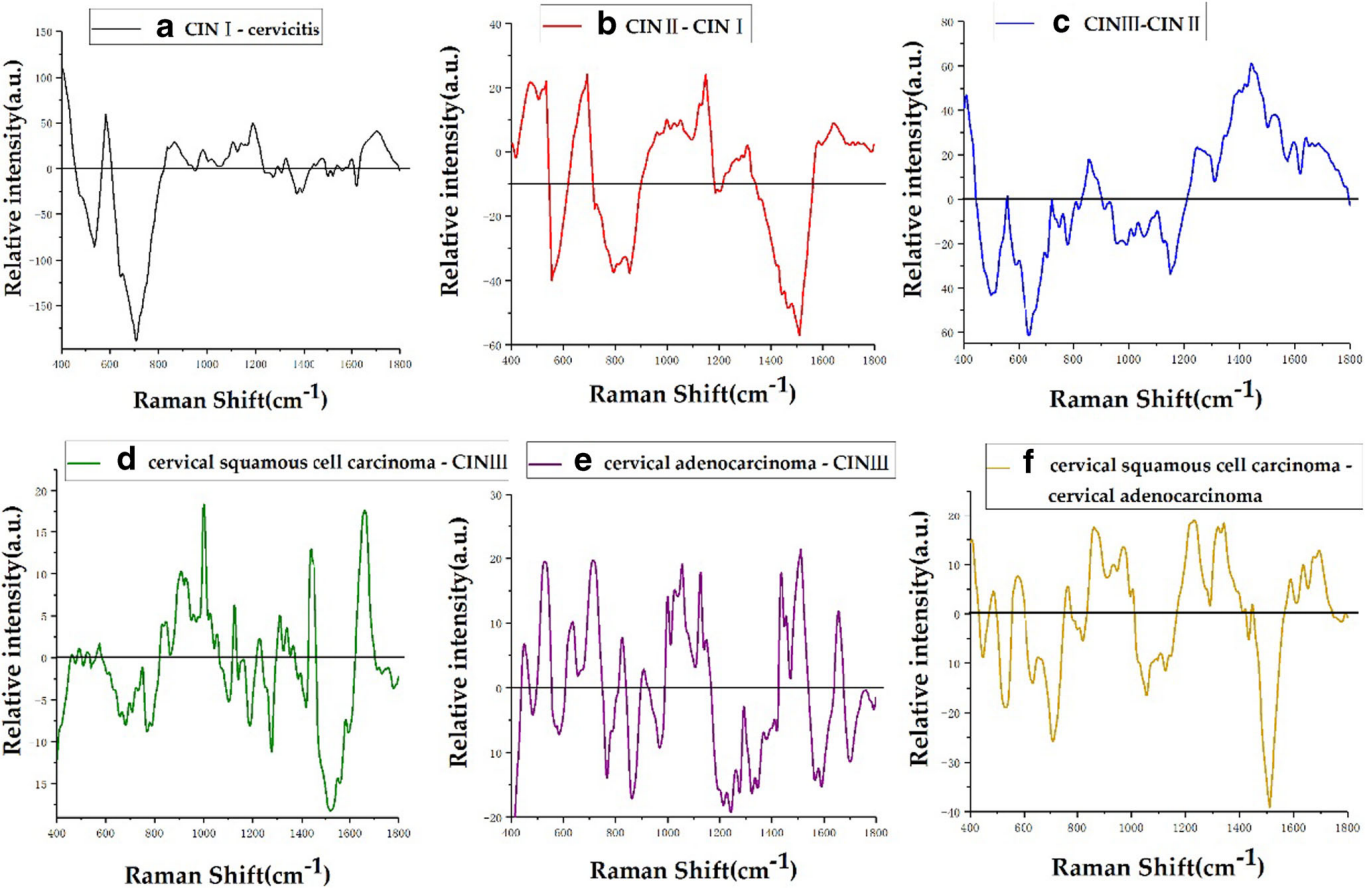
**a**–**f** Average Raman spectra of 6 types of cervical tissues based on the 1 to 1 difference spectra

For the convenience of viewing, the average spectral patterns of various types of cervical tissues in Fig. 2 were vertically translated. In order to directly reflect the relative intensity of different cervical tissue Raman characteristic peaks, Fig. 3 and Fig. 4 show the average Raman spectral difference spectra of six types of cervical tissues and the 1 to 1 difference spectra between the average Raman spectrum of the six types of cervical tissues. From Fig. 3 and Fig. 4, the differences between the average Ramen peaks of each of the various tissue samples can be easily observed and contrasted.

Table 1 lists the tentative assignments for the primary spectral bands according to the previous literatures [7, 12–19]. It can be observed from Fig. 3 that the Raman peaks belonging to lipids, amino acids, collagen, and cytosine at 519, 548, 643, 754, 817, 1369, 1506, and 1618 cm^−1^ are the most intense in inflamed cervical tissue. At 1002, 1170, and 1664 cm^−1^ belonging to phenylalanine, tyrosine, and amide I, the characteristic peaks of cervical inflammation tissue were the weakest. At 877 cm^−1^, a lipid-derived peak, the relative intensity of inflammatory tissue characteristic peaks is stronger than that of CIN I, CIN III, and cervical squamous cell carcinoma tissues, but weaker than that of CIN II and cervical adenocarcinoma tissues. At 951 cm^−1^, belonging to proteins (α-helix), the relative intensity of inflammatory tissue is stronger than that of CIN I, CIN III, cervical squamous cell carcinoma, and cervical adenocarcinoma, but weaker than that of CIN II. At 1239 cm^−1^, belonging to amide III, the relative intensity of the characteristic peak of inflammation tissue is stronger than that of CIN I, CIN II, and cervical adenocarcinoma, which is weaker than that of CIN III and cervical squamous cell carcinoma. At 1270 cm^−1^, belonging to phospholipids, the relative intensity of inflammatory tissue is stronger than that of CIN I, CIN II, and cervical adenocarcinoma and weaker than that of CIN III and cervical squamous cell carcinoma. At 1303 cm^−1^, belonging to collagen, the relative intensity of inflammatory tissue characteristic peaks is stronger than that of CIN I and CIN II tissue and weaker than that of CIN III, cervical squamous cell carcinoma, and cervical adenocarcinoma. At 1449 cm^−1^, belonging to CH vibration (proteins) and CH vibration (lipids), the relative intensity of inflammatory tissue characteristic peaks is weaker than that of CIN I, CIN III, cervical squamous cell carcinoma, and cervical adenocarcinoma and stronger than that of CIN II. At 1560 cm^−1^, belonging to tryptophan, the relative intensity of inflammatory tissue characteristic peaks was stronger than that of CIN I, CIN II, cervical squamous cell carcinoma, and cervical adenocarcinoma and weaker than that of CIN III tissue. The relative intensity of the Raman characteristic peaks can well reflect the concentration and content of the corresponding biochemical components in cervical inflammation tissue and cervical precancerous lesions (CIN I, CIN II, CIN III) and cervical squamous cell carcinoma. The difference in the relative intensity of the Raman characteristic peak between cancers reflects the obvious biochemical difference between the inflamed tissue and the other five types of cervical tissue, indicating that the biochemistry of cervical tissue changes throughout the development of cervical lesions.

**Table 1:** The peak positions and tentative assignments of the primary Raman bands

Raman peak (cm^−1^)	Assignment	References
477	Polysaccharides, amylose	[13]
481	DNA	[14]
495–516	Amino acid and cysteine	[13]
519	Phosphatidylinositol	[14–16]
538	Cholesterol ester	[14]
548	Cholesterol	[14–16]
576	Phosphatidylinositol	[14]
630	Glycerol	[14]
643	Tyrosine	[14–16]
754	Symmetric breathing of tryptophan (protein assignment)	[12, 17, 18]
766	Pyrimidine ring breathing mode	[14]
780	Uracil-based ring breathing mode	[14]
788	DNA	[14]
817	C–C stretching (collagen assignment)	[14–16]
826	DNA	[14]
830	Tyrosine	[13]
855	Proline, tyrosine	[15]
859	Tyrosine, collagen	[14]
867	Ribose vibration, one of the distinct RNA modes	[19]
877	Lipids	[14]
928	Proline, valine (protein band)	[14]
951	Proteins (α-helix)	[14]
968	Lipids	[18]
970	Phosphate monoester groups of phosphorylated proteins and cellular nucleic acids	[18]
1000	Phenylalanine	[17]
1002	Phenylalanine	[14]
1004	Phenylalanine (of collagen)	[14]
1025	Glycogen	[18]
1030	Phenylalanine of collagen	[14]
1053	C–O stretching, C–N stretching (protein)	[19]
1057	Lipids	[18]
1104	Phenylalanine (proteins)	[14]
1124	Lipids	14
1128	C–N stretching (proteins)	[14]
1150	Glycogen	[18]
1158	Proteins	[14]
1170	C–H in-plane bending mode of tyrosine	[14]
1230	Anti-symmetric phosphate stretching vibration	[18]
1243	Amide III	[13]
1246	Amide III (of collagen)	[14]
1275	Amide III	[13]
1290	Cytosine	[14]
1303	Collagen	[14]
1309	CH3/CH2 twisting or bending mode of lipid/collagen	[14]
1320	G (DNA/RNA)	[14]
1339	C–C stretch of phenylalanine	[14]
1365	Tryptophan	[14]
1369	Guanine, TRP (protein), porphyrins, lipids	[14–16]
1437	CH2 deformation (lipid)	[14]
1445	CH3/CH2 bending modes of collagen and phospholipids	[15]
1510	A (ring breathing modes in the DNA bases)	[19]
1560	Tryptophan	[14]
1583	Phenylalanine	[14]
1618	Tryptophan (protein assignment)	[14–16]
1637	Amide I band	[7]
1645	Amide I (α-helix)	[14]
1654	Amide I (collagen assignment)	[16]
1697	Amide I (turns and bands)	[14]
1185–300	Anti-symmetric phosphate vibrations	[12]
1437–53	CH2 deformation	[14]
1506	Cytosine	[12, 17, 18]
1520–38	Carotenoid	[14]
1588	Phenylalanine, hydroxyproline	[14]
1600–800	Amide I	[19]
1640	Amide I band (protein band)	[14]
1655–80	Amide I (proteins)	[14]
1664	Amide I	[14]
1700–50	Amino acids aspartic acid and glutamic acid	[13]

Figure 4b shows the difference spectra of CIN II-CIN I. The positive peaks at 477, 538, 692, and 1000 cm^−1^ are attributable to polysaccharides, cholesterol ester, amino acids, and methionine, respectively. Among phenylalanine peaks, 1030 cm^−1^ is associated with collagen, 1053 and 1128 cm^−1^ belong to protein, 1150 cm^−1^ belongs to glycogen, 1290 cm^−1^ belongs to cytosine, 1313 cm^−1^ corresponds to the twisting mode of collagen and lipids, 1583 cm^−1^ belongs to phenylalanine, and 1645 cm^−1^ belongs to amide I (α-helix). The negative peaks mainly appear at 788 cm^−1^ (DNA), 855 cm^−1^ (proline, tyrosine), and 1510 cm^−1^ (ring breathing modes in the DNA bases). Figure 4c shows the difference spectra of CIN III-CIN II. It can be seen that the positive peaks mainly appear at 859 cm^−1^, belonging to tyrosine and collagen; 1246 cm^−1^, attributed to amide III (of collagen); and 1445 cm^−1^, attributed to CH3 and CH2 bending modes of collagen and phospholipids. In the examination of the bending modes of collagen and phospholipids, we found that 1520–38 cm^−1^ belongs to carotenoid, 1600–800 cm^−1^ belongs to amide I, and 1640 cm^−1^ belongs to amide I (protein band). The negative peaks mainly appear at 495–516 cm^−1^ belonging to amino acid and cysteine, 780 cm^−1^ is associated with uracil-based ring breathing mode, and 1158 cm^−1^ is associated with proteins. Figure 4d shows difference spectra of cervical squamous cell carcinoma-CIN III. It can be seen that the positive peaks are mainly at 830 cm^−1^, belonging to tyrosine; 928 cm^−1^, corresponding to amino acids; and 1000 cm^−1^, belonging to phenylalanine. Additionally, 1025 cm^−1^ is attributable to glycogen, 1053 cm^−1^ belongs to protein, 1309 cm^−1^ belongs to the CH3/CH2 twisting or bending mode of lipid/collagen, 1339 cm^−1^ belongs to CC stretch of phenylalanine, 1365 cm^−1^ belongs to tryptophan, 1437–53 cm^−1^ belongs to CH2 deformation of lipid, and 1655–80 cm^−1^ belongs to amide I (proteins); negative peaks mainly appear at 766 cm^−1^ belonging to the pyrimidine ring breathing mode, 1104 cm^−1^ belonging to phenylalanine (proteins), 1185–300 cm^−1^ belonging to anti-symmetric phosphate vibrations, and 1275 cm^−1^ belonging to amide III. Figure 4e shows difference spectra of cervical adenocarcinoma-CIN III. It can be seen that the positive peak is mainly at 447/54 cm^−1^ at ring torsion of phenylalanine, 634 cm^−1^ attributed to amino acid and methionine, 826 cm^−1^ belonging to DNA, 1025 cm^−1^ belonging to glycogen, 1057 cm^−1^ belonging to lipids, 1437 cm^−1^ belonging to CH2 deformation (lipid), 1510 cm^−1^ belonging to ring breathing modes in the DNA bases, and 1654 cm^−1^ belonging to amide I (collagen assignment); the negative peak mainly appears at 481 cm^−1^ belonging to DNA, 766 cm^−1^ belonging to pyrimidine ring breathing mode, 968 cm^−1^ belonging to lipids, 1243 cm^−1^ belonging to amide III, 1560 cm^−1^ belonging to tryptophan, and 1700–50 cm^−1^ belonging to amino acids aspartic acid and glutamic acid. Figure 3f shows difference spectra of cervical squamous cell carcinoma-cervical adenocarcinoma. It can be seen that the positive peak mainly occurs at 576 cm^−1^ belonging to phosphatidylinositol, 766 cm^−1^ belonging to the pyrimidine ring breathing mode, and 867 cm^−1^ belonging to ribose vibration. Of the distinct RNA modes, 970 cm^−1^ belongs to phosphate monoester groups of phosphorylated proteins and cellular nucleic acids, 1004 cm^−1^ belongs to phenylalanine (of collagen), 1230 cm^−1^ belongs to anti-symmetric phosphate stretching vibration, 1320 cm^−1^ belongs to G (DNA/RNA), 1588 cm^−1^ belongs to phenylalanine and hydroxyproline, 1637 cm^−1^ belongs to the amide I band, and 1697 cm^−1^ belongs to amide I (turns and bands). The negative peaks are mainly found in 630 cm^−1^, belonging to glycerol; 1057 cm^−1^, corresponding to lipids; 1124 cm^−1^, associated with lipids; and 1510 cm^−1^, belonging to A (ring breathing modes in the DNA bases).

There is a significant difference between the mean Raman spectra of the six types of cervical tissues, and these differences can be used to identify the six types of cervical tissues. Then, SVM was used to establish a classification model for the six types of cervical tissues.

### Statistical analysis results

Table 2 lists the independent sample *t* test of the average relative intensity of six kinds of cervical tissue Raman spectra. And Table 3 lists the relative intensities of representative characteristic peaks of lipids, proteins, and nucleic acids in six types of cervical tissues.

**Table 2:** Independent sample *t* test of the average relative intensity of six kinds of cervical tissue Raman spectra

Cervical tissue type	*p* value
Cervicitis vs. CIN I	0.001
Cervicitis vs. CIN II	0.000
Cervicitis vs. CIN III	0.000
Cervicitis vs. cervical squamous cell carcinoma	0.000
Cervicitis vs. cervical adenocarcinoma	0.000
CIN I vs. CIN II	0.000
CIN I vs. CIN III	0.001
CIN I vs. cervical squamous cell carcinoma	0.000
CIN I vs. cervical adenocarcinoma	0.000
CIN II vs. CIN III	0.002
CIN II vs. cervical squamous cell carcinoma	0.046
CIN II vs. cervical adenocarcinoma	0.020
CIN III vs. cervical squamous cell carcinoma	0.282
CIN III vs. cervical adenocarcinoma	0.435
Cervical squamous cell carcinoma vs. cervical adenocarcinoma	0.762

**Table 3:** The relative intensities of representative characteristic peaks of lipids (877 cm^−1^), proteins (1002 cm^−1^), and nucleic acids (1510 cm^−1^) in six types of cervical tissues

Cervical tissue type	Relative intensity (877 cm^−1^)	Relative intensity (1002 cm^−1^)	Relative intensity (1510 cm^−1^)
Cervicitis	− 6.82	8.67	2.62
CIN I	18.56	17.06	0.19
CIN II	− 7.34	26.97	− 22.04
CIN III	2.87	11.23	− 56.93
Cervical squamous cell carcinoma	5.18	29.22	− 39.59
Cervical adenocarcinoma	− 11.06	24.49	− 1.03

There were significant differences in the average Raman spectra of the 6 types of cervical tissues, which could be used to identify the 6 types of cervical tissues. In this study, six types of cervical tissues were classified by SVM.

In this study, the Raman spectra of six types of cervical lesions were analyzed comprehensively for the first time, and the six types of cervical lesions were classified by Raman spectroscopy combined with support vector machine. The svmtrain function is used to build the classification model, and the function parameters are set to “-t 1 -c 2 -g 0.02”, where t represents the type of kernel function and c and g represent penalty parameter and kernel parameter, respectively. After repeated experiments, the classification effect is better.

A total of 1110 original Raman spectra of cervical tissue were used in the SVM classification model (293 spectra of cervical inflammation tissue, 157 spectra of CIN I tissue, 138 spectra of CIN II tissue, 155 spectra of CIN III tissue, 166 spectra of cervical squamous cell carcinoma, and 201 spectra of cervical adenocarcinoma). Thirty-five spectra were randomly selected from the original Raman spectra of various cervical tissues as test set data for modeling, and the remaining 900 spectra were used as training set data. The correct rate of classification is shown in Table 4, and the overall accuracy rate is 85.7%, based on the established SVM classification model, which indicated that Raman spectroscopy combined with support vector machine could be used to successfully classify cervical inflammation, cervical precancerous lesions (CIN I, CIN II, CIN III), and cervical cancer (cervical squamous cell carcinoma, cervical adenocarcinoma).

**Table 4:** Classification results of SVM algorithm

Style polynomial	*N*(train)/*N*(test)	Correct	Fault	Total	Accuracy
Cervicitis	258/35	27	8	293	77.14%
CIN I	122/35	31	4	157	88.57%
CIN II	103/35	28	7	138	80%
CIN III	120/35	29	6	155	82.86%
Cervical squamous cell carcinoma	131/35	35	0	166	100%
Cervical adenocarcinoma	166/35	30	5	201	85.71%

## Discussion

In this study, a confocal Raman micro-spectrometer was used to analyze 6 types of cervical tissues (cervical inflammation, CIN I, CIN II, CIN III, cervical squamous cell carcinoma, and cervical adenocarcinoma), and a total of 1110 Raman spectra were obtained. The average Raman spectra of six types of cervical tissues (Fig. 2), the difference spectra of average Raman spectra (Fig. 3), and the difference spectra of average Raman spectra of one to one (Fig. 4) were drawn, compared, and analyzed. The main Raman characteristic peaks of six types of cervical tissues were summarized, and the differences of biochemical components of six types of cervical tissues were analyzed. This study reveals that with the development of cervical lesions (cervical inflammation → CIN I → CIN II → CIN III → cervical cancer), the biochemical components of cervical tissues are also changing. It also reveals that there are significant differences in biochemical components between cervical squamous cell carcinoma and cervical adenocarcinoma.

## Conclusion

It was concluded that Raman spectroscopy combined with SVM can successfully classify cervicitis, cervical precancerous lesions (CIN I, CIN II, CIN III), cervical cancer (cervical squamous cell carcinoma, cervical adenocarcinoma), and the accuracy of six cervical tissues is 85.7%. This study is expected to provide a new method for the clinical diagnosis of cervical lesions.
